# Supplementary material for: Structural Pockets and Interacting RNA-Associated Ligands (SPIRAL): A DSSR-enabled Meta-Analysis of RNA-Small Molecule Recognition
Source: bioRxiv. 2026 Jun 23:2026.05.19.726393. Originally published 2026 May 21. Preprint. [Version 2] doi: 10.64898/2026.05.19.726393 (PMC13228370; doi:10.64898/2026.05.19.726393)
Supplement: Supplement 1 [file media-1.pdf]

Supporting information

Structural Pockets and Interacting RNA-Associated Ligands (SPIRAL): A DSSR-enabled Meta-Analysis of RNA-Small Molecule Recognition

Xiang-Jun Lu[1,*] and Yaqiang Wang[2,3,4,*]

[1] Department of Biological Sciences, Columbia University, New York, NY 10027, United States
[2] Department of Biophysics, Medical College of Wisconsin, Milwaukee, WI 53226, United States
[3] Department of Obstetrics and Gynecology, Medical College of Wisconsin, Milwaukee, WI 53226, United States
[4] Medical College of Wisconsin Cancer Center, Milwaukee, WI 53226, United States

* To whom correspondence should be addressed. Email: yawang@mcw.edu
Correspondence may also be addressed to Xiang-Jun Lu. Email: xiangjun@x3dna.org

Figure S1

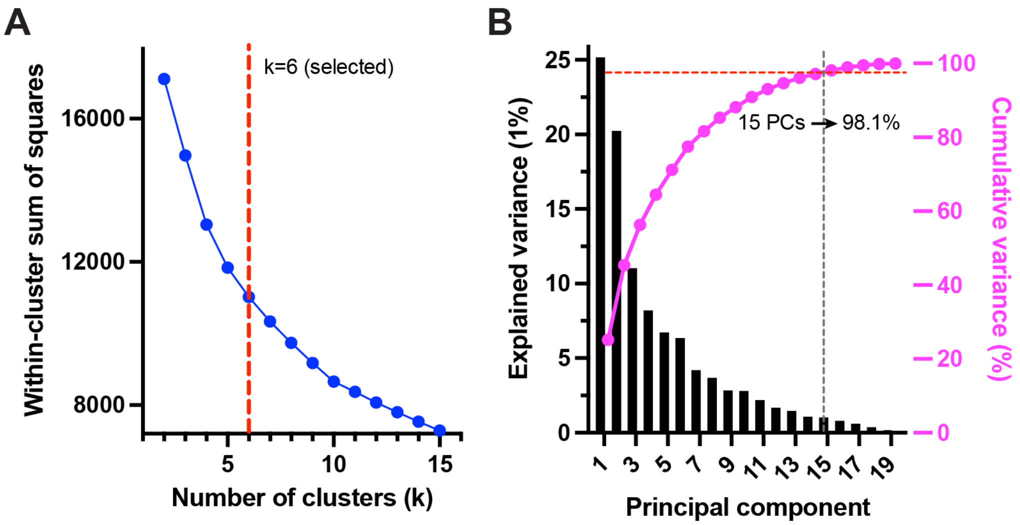

Figure S1 Elbow analysis for k selection. A) Elbow analysis. Within-cluster sum of squares plotted for k = 2 to 15, with k = 6 marked by a dashed red line. B) PCA explained variance: individual component variance (19 components) shown as bars with cumulative variance as a red overlay line, with the 98.1% threshold marked, confirming that 15 components capture 98.1% of variance with negligible information loss.

Figure S2

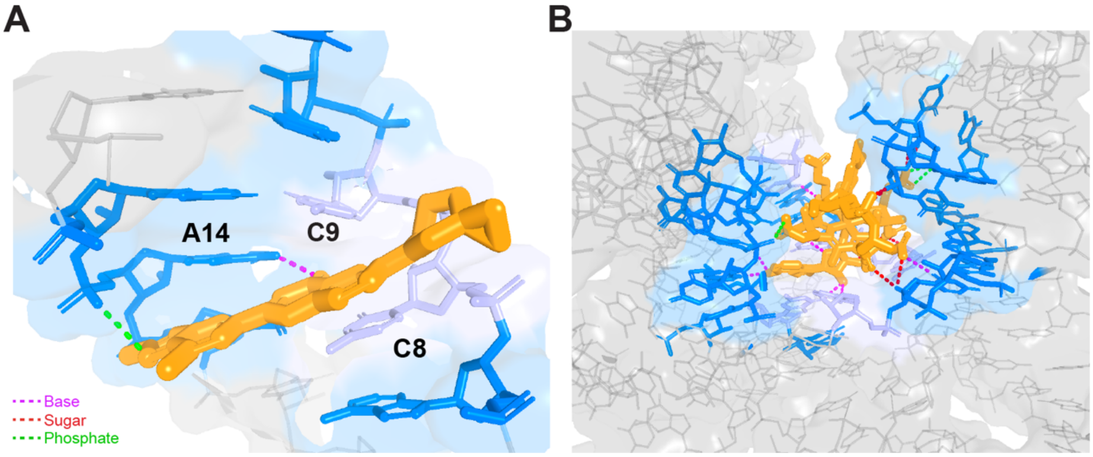

Figure S2. Representative RNA–small molecule structures illustrating DSSR-computed interaction parameters extracted by SPIRAL. A) Risdiplam (PDB ID: 8R62) bound to the SMN2 pre-mRNA 5′-splice site duplex. Its binding mode exemplifies the major groove recognition class: the ligand interacts with two cytosine residues (C8 and C9) within the RNA stem. Only two hydrogen bonds are formed: one to the base of A14 (N6) and one to its phosphate (OP2), while no 2′-OH contacts are detected, and the binding site is classified as a major groove binder. B) Adenosylcobalamin (PDB ID: 4GMA) bound to the adenosylcobalamin riboswitch. The large corrin ring system is enclosed by 22 contacted nucleotides spanning junction loops, hairpin loops, a pseudoknot, and kissing-loop elements, with a total buried contact surface area of 1867 Å^2. Five nucleotides stack on the corrin ring, and 17 hydrogen bonds are distributed across all three RNA moiety classes: six to nucleobases, five to phosphate oxygens, and six to ribose 2′-OH groups. In both panels, the full RNA is shown as faded grey sticks with a transparent grey surface; interface nucleotides are marine; stacking nucleotides are light blue; the ligand is orange; hydrogen bonds to bases, phosphate oxygens, and ribose 2′-OH groups are shown as magenta, green, and red dashes, respectively.

Figure S3

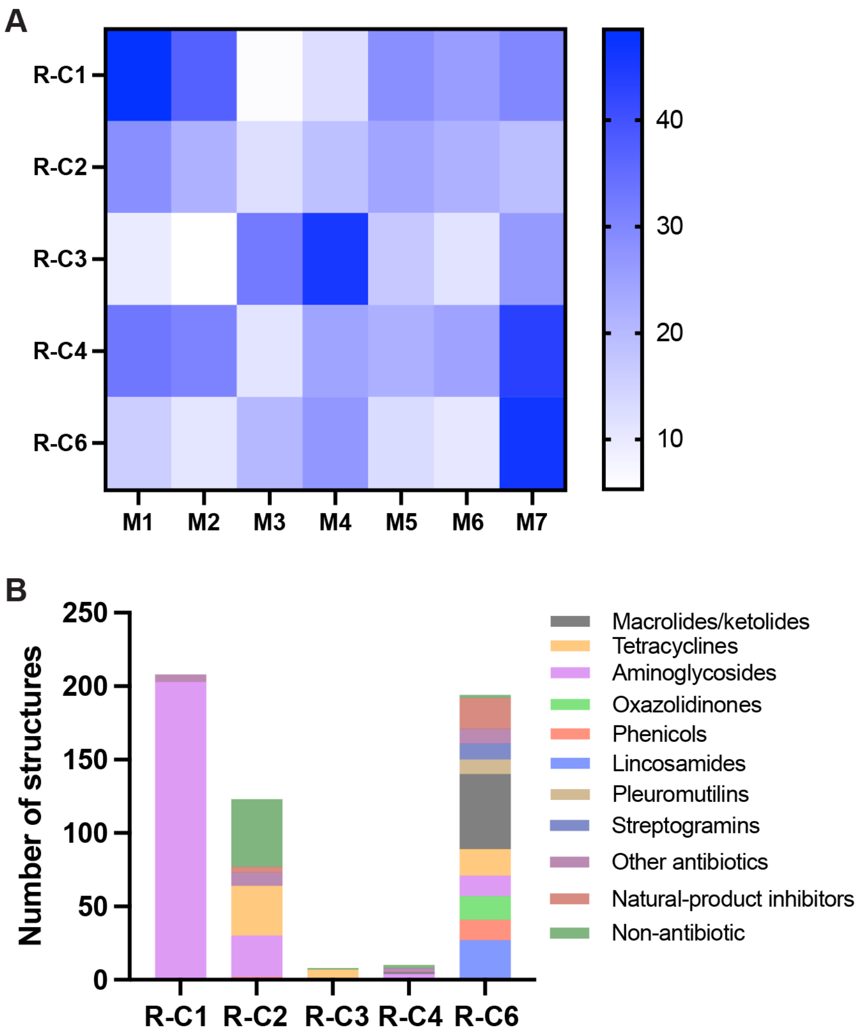

Figure S3 Interaction profiles and drug class distribution across five ribosome RNA binding mode sub-sites. A) Heatmap of interaction parameter profiles for the five clusters containing ribosome RNA entries (R-C1, R-C2, R-C3, R-C4, and R-C6; C5 is excluded as it contains no ribosome RNA entries). Rows represent sub-sites sorted by entry count; columns represent seven CBQS sub-scores (M1–M7); color intensity reflects the normalized score on a 0–50 scale. B) Approximate drug class distribution across the five ribosome sub-sites, expressed as percentage of ligand-binding events per cluster. R-C1 is dominated by aminoglycosides (98%), corresponding to the 30S decoding A-site. R-C6 groups 50S peptidyl-transferase-center and exit-tunnel antibiotics, including macrolides and ketolides (26%), lincosamides (14%), and oxazolidinones (8%). R-C2 is a mixed cluster combining non-antibiotic ligands (37%), tetracyclines (28%), and aminoglycosides (23%). R-C3 is enriched for tetracyclines (75%).

Table S1 Summary of the predefined exclusion list applied during SPIRAL dataset curation

Functional category	Amount	CCD codes
Metal ions and elemental atoms	28	BA, BR, C, CA, CD, CL, CO, 3CO, CS, CU1, F, HG, IRI, K, LU, MG, MN, N, NA, NH4, O, OS, PB, RHD, SR, TL, W, ZN
Inorganic ions and clusters	14	FES, FS2, NCO, NO3, OHX, PO4, POP, SE4, SF4, SO4, VTR, VTE, WO2, WO4
PEG fragments	11	1PE, 2PE, 4PE, 5PE, 6PE, 7PE, 8PE, P6G, PEG, PG4, PGE
Buffer components	14	ACE, ACT, ACY, 3TD, BTB, CAC, EPE, GZ6, MES, MLI, N2P, N3D, TLA, TRS
Cryoprotectants and solvents	11	BME, DMS, EDO, EOH, GOL, HEZ, IPA, IPH, MPD, PDI, TER
Other crystallization additives	15	2HP, B3P, CPT, DPO, GAI, GDE, IHP, NME, PUT, S9L, SPD, SPK, SPM, TAM, URE
Non-pharmacological cofactors	3	ARF, FME, SIN
Nucleotide-related compounds	10	8OS, 50L, 50N, A1CGJ, EQ1, EQ4, G3A, GP3, LXI, LXR
Amino acids	7	ARG, GLY, LYS, MET, PHE, PRO, TYR
Other non-drug-like biomolecules	5	6MZ, CAD, FRU, GLC, SEY
Unidentified PDB placeholders	2	UNL, UNX
Total	120	

Table S2 Functional RNA PDB category assignment criteria for the SPIRAL dataset

Category	Amount*	%	Keywords	Rfam family
G-quadruplexes	50	4.6%	quadruplex, G4, G-tetrad, G-quartet, fluorescent, dimer, biotin, hexammine, iridium, Spinach, Mango, Corn, Beetroot, Chili	Not Rfam-classified (structural class)
Regulatory motifs	67	6.1%	TAR, bulge, HIV, interaction, hairpin, stem-loop, solution NMR	RF00250 (HIV-1 TAR); and other functional RNA motif families
Ribosome	512	46.6%	ribosomal, subunit, rRNA, ribosome, translation, mitochondrial, cytosolic	RF00177 (small subunit rRNA); RF02543 (large subunit rRNA); RF00001 (5S rRNA)
Riboswitches	368	33.5%	riboswitch, aptamer, domain, variant, mutant, ligand-gated	RF00050 (FMN); RF00059 (TPP); RF00162 (SAM); RF00167 (purine); RF00174 (cobalamin); RF01739 (glutamine); RF01786 (c-di-GMP); RF00522 (preQ1); RF01734 (fluoride)
Ribozymes	38	3.5%	ribozyme, snRNP, splicing, substrate, spliceosome, self-splicing, ribonucleoprotein, glmS	RF00163 (hammerhead); RF00173 (hairpin); RF01577 (RNase P); RF02001 (group II intron)
Synthetic aptamers	63	5.7%	aptamer, Fab, chain, heavy, light, theophylline, soak, SELEX, Pepper, Squash, RhoBAST	Not Rfam-classified (in vitro selected constructs)
Total	1098	100%		

*Amount = PDB entries (structures), whereas n values in main text are ligand binding events.

Table S3 Statistical analyses of cluster and category comparisons

Comparison	Test	Statistic	n	p / q value	Sidedness	Significant (α=0.05)
Cluster identity × RNA category	Chi-square test of independence	χ^2 = 1428.4, df = 25	1137	3.32e-286	two-sided	Yes
Overall CBQS across 6 categories	Kruskal–Wallis omnibus	H = 258.4	1137	8.60e-54	two-sided	Yes
H-bond quality (M1) across 6 categories	Kruskal–Wallis omnibus	H = 146.4	1137	7.68e-30	two-sided	Yes
Stacking density (M4) across 6 categories	Kruskal–Wallis omnibus	H = 422.0	1137	5.45e-89	two-sided	Yes
Pocket complexity (M7) across 6 categories	Kruskal–Wallis omnibus	H = 218.6	1137	3.03e-45	two-sided	Yes
Riboswitches vs Ribosome (CBQS)	Mann–Whitney U (BH-FDR)	U = 156915	370 / 543	q < 0.001	two-sided	Yes
Riboswitches vs Regulatory motifs (CBQS)	Mann–Whitney U (BH-FDR)	U = 21306	370 / 67	q < 0.001	two-sided	Yes
Riboswitches vs G-quadruplexes (CBQS)	Mann–Whitney U (BH-FDR)	U = 14223	370 / 51	q < 0.001	two-sided	Yes
Riboswitches vs Synthetic aptamers (CBQS)	Mann–Whitney U (BH-FDR)	U = 17352	370 / 66	q < 0.001	two-sided	Yes
Ribozymes vs Regulatory motifs (CBQS)	Mann–Whitney U (BH-FDR)	U = 2188	40 / 67	q < 0.001	two-sided	Yes
Synthetic aptamers vs Regulatory motifs (CBQS)	Mann–Whitney U (BH-FDR)	U = 3433	66 / 67	q < 0.001	two-sided	Yes
Ribozymes vs Ribosome (CBQS)	Mann–Whitney U (BH-FDR)	U = 15404	40 / 543	q < 0.001	two-sided	Yes
Ribosome vs Regulatory motifs (CBQS)	Mann–Whitney U (BH-FDR)	U = 23654	543 / 67	q < 0.001	two-sided	Yes
G-quadruplexes vs Regulatory motifs (CBQS)	Mann–Whitney U (BH-FDR)	U = 2373	51 / 67	q < 0.001	two-sided	Yes
Ribozymes vs G-quadruplexes (CBQS)	Mann–Whitney U (BH-FDR)	U = 1395	40 / 51	q = 0.004	two-sided	Yes

Comparison	Test	Statistic	n	p / q value	Sidedness	Significant (α=0.05)
Synthetic aptamers vs Ribosome (CBQS)	Mann–Whitney U (BH-FDR)	U = 21950	66 / 543	q = 0.004	two-sided	Yes
Ribozymes vs Synthetic aptamers (CBQS)	Mann–Whitney U (BH-FDR)	U = 1650	40 / 66	q = 0.040	two-sided	Yes
Riboswitches vs Ribozymes (CBQS)	Mann–Whitney U (BH-FDR)	U = 8743	370 / 40	q = 0.068	two-sided	No
Synthetic aptamers vs G-quadruplexes (CBQS)	Mann–Whitney U (BH-FDR)	U = 1950	66 / 51	q = 0.153	two-sided	No
G-quadruplexes vs Ribosome (CBQS)	Mann–Whitney U (BH-FDR)	U = 15074	51 / 543	q = 0.295	two-sided	No

Symbols: χ^2, chi-square statistic; df, degrees of freedom; H, Kruskal–Wallis statistic; U, Mann–Whitney U statistic; q, Benjamini–Hochberg FDR-adjusted p value; α, significance threshold. All tests are two-sided. For pairwise comparisons, n is given as the size of each group (group A / group B). Chi-square evaluated cluster identity against RNA functional category across 1,137 binding events; Kruskal–Wallis tests compared each interaction metric across the six categories; pairwise category comparisons used Mann–Whitney U tests with Benjamini–Hochberg correction. Analyses were performed in Python using scipy and statsmodels.

Table S4 Affinity correlation statistics (n = 275 affinity-characterized entries)

Analysis	Variable / subgroup	Spearman ρ	95% CI	n	p value	Significant (α=0.05)
Global predictors	C2′-endo pucker count	+0.530	[+0.44, +0.61]	275	2.49e-21	Yes
Global predictors	Buried contact area (Å²)	+0.440	[+0.34, +0.53]	275	1.76e-14	Yes
Global predictors	H-bond count	-0.016	[-0.13, +0.10]	275	7.88e-01	No
Simpson's paradox (H-bond vs pKd)	Global (pooled)	-0.016	[-0.13, +0.10]	275	0.788	No
Simpson's paradox (H-bond vs pKd)	Riboswitches	+0.384	[+0.25, +0.51]	169	2.5e-07	Yes
Simpson's paradox (H-bond vs pKd)	Synthetic aptamers	-0.241	[-0.53, +0.09]	36	0.156	No
Simpson's paradox (H-bond vs pKd)	G-quadruplexes	-0.444	[-0.69, -0.10]	30	0.014	Yes
Simpson's paradox (H-bond vs pKd)	Ribosome RNA	+0.544	[+0.04, +0.83]	15	0.036	Yes
Simpson's paradox (H-bond vs pKd)	Regulatory motifs	+0.122	[-0.30, +0.50]	24	0.571	No

Symbols: ρ, Spearman rank correlation coefficient; CI, confidence interval. All correlations are two-sided Spearman rank correlations; 95% confidence intervals were computed by Fisher z-transformation. Ribozymes (n = 1 affinity entry) were not estimable and are omitted. A two-predictor linear regression model (buried contact area and C2′-endo pucker count, RobustScaler-normalized) achieved a mean five-fold cross-validated R^2 of 0.40. Analyses were performed in Python using scipy and scikit-learn.
