# Supplementary material for: Structural Pockets and Interacting RNA-Associated Ligands (SPIRAL): A DSSR-enabled Meta-Analysis of RNA-Small Molecule Recognition
Source: bioRxiv. 2026 May 21:2026.05.19.726393. Preprint. [Version 1] doi: 10.64898/2026.05.19.726393 (PMC13228370; doi:10.64898/2026.05.19.726393)
Supplement: Supplementary file 1 [file media-1.pdf]

Supporting information

Structural Pockets and Interacting RNA-Associated Ligands (SPIRAL): A DSSR-enabled Meta-Analysis of RNA-Small Molecule Recognition

Xiang-Jun Lu[1,*] and Yaqiang Wang[2,3,4,*]

[1] Department of Biological Sciences, Columbia University, New York, NY 10027, United States
[2] Department of Biophysics, Medical College of Wisconsin, Milwaukee, WI 53226, United States
[3] Department of Obstetrics and Gynecology, Medical College of Wisconsin, Milwaukee, WI 53226, United States
[4] Medical College of Wisconsin Cancer Center, Milwaukee, WI 53226, United States

* To whom correspondence should be addressed. Email: yawang@mcw.edu
Correspondence may also be addressed to Xiang-Jun Lu. Email: xiangjun@x3dna.org

Figure S1

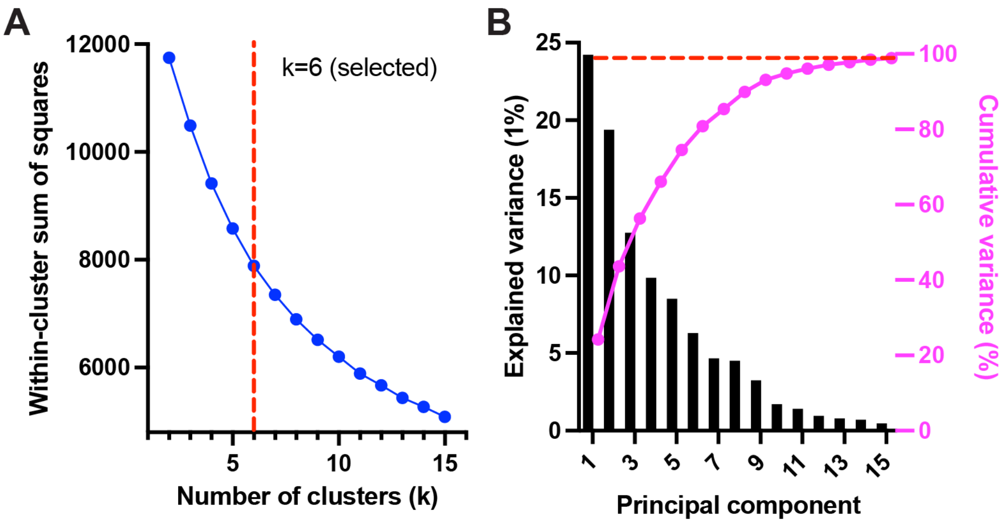

Figure S1 Elbow analysis for k selection. A) Elbow analysis. Within-cluster sum of squares plotted for k = 2 to 15, with k = 6 marked by a dashed red line. B) PCA explained variance: Individual component variance as bars with cumulative variance as a red overlay line, with the 99.1% threshold marked, confirming that 15 components capture 98.9% of variance with negligible information loss.

Figure S2

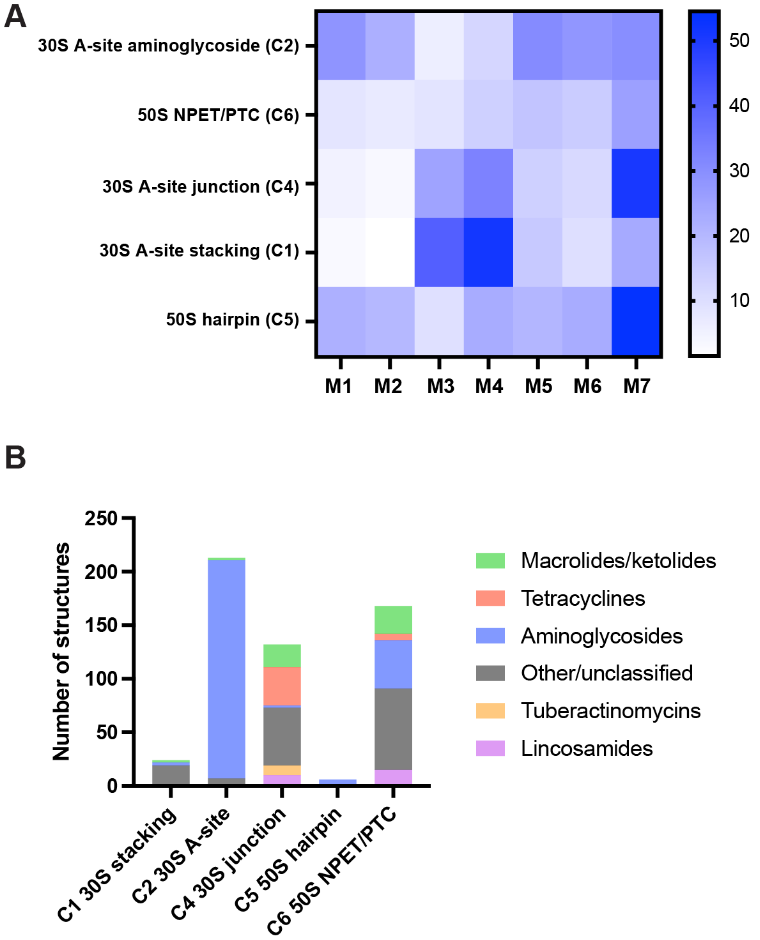

Figure S2 Interaction profiles and drug class distribution across five ribosome RNA binding mode sub-sites. A) Heatmap of interaction parameter profiles for the five clusters containing ribosome RNA entries (C1, C2, C4, C5, and C6; C3 is excluded as it contains no ribosome RNA entries). Rows represent sub-sites sorted by entry count; columns represent seven CBQS sub-scores (M1–M7); color intensity reflects the normalized score on a 0–55 scale. B) Approximate drug class distribution across the five ribosome sub-sites, expressed as percentage of ligand-binding events per cluster. C2 is dominated by aminoglycosides (96%). C4 is enriched for tetracyclines (27%) and tuberactinomycins (7%), alongside a substantial proportion of novel experimental compounds (41%). C6 groups macrolides and ketolides (15%) with lincosamides (9%). The Other/unclassified category includes novel experimental antibiotics, research probes, nucleotide analogs, and ligands without established pharmacological classification.

Table S1 Summary of the predefined exclusion list applied during SPIRAL dataset curation

Functional category	Amount	CCD codes
Metal ions and elemental atoms	28	BA, BR, C, CA, CD, CL, CO, 3CO, CS, CU1, F, HG, IRI, K, LU, MG, MN, N, NA, NH4, O, OS, PB, RHD, SR, TL, W, ZN
Inorganic ions and clusters	14	FES, FS2, NCO, NO3, OHX, PO4, POP, SE4, SF4, SO4, VTR, VTE, WO2, WO4
PEG fragments	11	1PE, 2PE, 4PE, 5PE, 6PE, 7PE, 8PE, P6G, PEG, PG4, PGE
Buffer components	14	ACE, ACT, ACY, 3TD, BTB, CAC, EPE, GZ6, MES, MLI, N2P, N3D, TLA, TRS
Cryoprotectants and solvents	11	BME, DMS, EDO, EOH, GOL, HEZ, IPA, IPH, MPD, PDI, TER
Other crystallization additives	15	2HP, B3P, CPT, DPO, GAI, GDE, IHP, NME, PUT, S9L, SPD, SPK, SPM, TAM, URE
Non-pharmacological cofactors	3	ARF, FME, SIN
Nucleotide-related compounds	10	8OS, 50L, 50N, A1CGJ, EQ1, EQ4, G3A, GP3, LXI, LXR
Amino acids	7	ARG, GLY, LYS, MET, PHE, PRO, TYR
Other non-drug-like biomolecules	5	6MZ, CAD, FRU, GLC, SEY
Unidentified PDB placeholders	2	UNL, UNX
Total	120	

Table S2 Functional RNA PDB category assignment criteria for the SPIRAL dataset

Category	Amount	%	Keywords	Rfam family
G-quadruplexes	50	4.6%	quadruplex, G4, G-tetrad, G-quartet, fluorescent, dimer, biotin, hexammine, iridium, Spinach, Mango, Corn, Beetroot, Chili	Not Rfam-classified (structural class)
Regulatory motifs	67	6.1%	TAR, bulge, HIV, interaction, hairpin, stem-loop, solution NMR	RF00250 (HIV-1 TAR); and other functional RNA motif families
Ribosome	512	46.6%	ribosomal, subunit, rRNA, ribosome, translation, mitochondrial, cytosolic	RF00177 (small subunit rRNA); RF02543 (large subunit rRNA); RF00001 (5S rRNA)
Riboswitches	368	33.5%	riboswitch, aptamer, domain, variant, mutant, ligand-gated	RF00050 (FMN); RF00059 (TPP); RF00162 (SAM); RF00167 (purine); RF00174 (cobalamin); RF01739 (glutamine); RF01786 (c-di-GMP); RF00522 (preQ1); RF01734 (fluoride)
Ribozymes	38	3.5%	ribozyme, snRNP, splicing, substrate, spliceosome, self-splicing, ribonucleoprotein, glmS	RF00163 (hammerhead); RF00173 (hairpin); RF01577 (RNase P); RF02001 (group II intron)
Synthetic aptamers	63	5.7%	aptamer, Fab, chain, heavy, light, theophylline, soak, SELEX, Pepper, Squash, RhoBAST	Not Rfam-classified (in vitro selected constructs)
Total	1098	100%		
